# Improving Biomedical Knowledge Graph Quality: A Community Approach

**Published:** 2025-08-29

**Authors:** Katherina G Cortes, Shilpa Sundar, Sarah Gehrke, Keenan Manpearl, Junxia Lin, Daniel Robert Korn, Harry Caufield, Kevin Schaper, Justin Reese, Kushal Koirala, Lawrence E Hunter, E. Kathleen Carter, Marcello DeLuca, Arjun Krishnan, Chris Mungall, Melissa Haendel

**Affiliations:** 1 Department of Biomedical Informatics, University of Colorado Anschutz Medical Campus; 2 Carolina Health Informatics Program, University of North Carolina at Chapel Hill; 3 TISLab, Department of Genetics, University of North Carolina at Chapel Hill; 4 Environmental Genomics and Systems Biology, Lawrence Berkeley National Laboratory; 5 Curriculum in Bioinformatics and Computational Biology, University of North Carolina at Chapel Hill; 6 Department of Pediatrics, University of Chicago; 7 Renaissance Computing Institute, University of North Carolina at Chapel Hill; 8 Eshelman School of Pharmacy, University of North Carolina at Chapel Hill

## Abstract

Biomedical knowledge graphs (KGs) are widely used across research and translational settings, yet their design decisions and implementation are often opaque. Unlike ontologies that more frequently adhere to established creation principles, biomedical KGs lack consistent practices for construction, documentation, and dissemination. To address this gap, we introduce a set of evaluation criteria grounded in widely accepted data standards and principles from related fields. We apply these criteria to 16 biomedical KGs, revealing that even those that appear to align with best practices often obscure essential information required for external reuse. Moreover, biomedical KGs, despite pursuing similar goals and ingesting the same sources in some cases, display substantial variation in models, source integration, and terminology for node types. Reaping the potential benefits of knowledge graphs for biomedical research while reducing duplicated effort requires community-wide adoption of shared criteria and maturation of standards such as Biolink and KGX. Such improvements in transparency and standardization are essential for creating long-term reusability, improving comparability across resources, providing a rigorous foundation for artificial intelligence models, and enhancing the overall utility of KGs within biomedicine.

## Background and Previous Work

Knowledge graphs (KGs) have gained popularity in recent years for their ability to connect different types and sources of data, offering a powerful computational framework to uncover novel biological hypotheses. Biomedical KG entities consist of disease, gene, mechanism, and other biological types. “Properties” linking entities include relationships such as ‘expressed in,’ ‘has phenotype’, or ‘interacts with.’ Biomedical KGs are typically constructed by aggregating and harmonizing existing curated knowledge bases and ontologies, supplemented by additional information that may come from text mining, data analyses, or case-level information. By ingesting and harmonizing many sources of knowledge, KGs are able to connect knowledge across biological domains leveraging existing research. For instance, the ROBOKOP (Reasoning Over Biomedical Objects linked in Knowledge Oriented Pathways)[1,2] KG platform enables researchers to query complex biomedical questions, such as “What environmental exposures are associated with asthma through intermediate biological processes?”, by integrating data from curated databases like CTD, DrugBank, and gene ontology (GO) annotations [3–5]. KGs have gained particular traction in biomedical research domains including drug repurposing, disease mechanism discovery, and rare disease illumination. Previous approaches have included using embeddings, vector representations of the nodes and edges which can then be used as input for artificial intelligence (AI) algorithms, of these KGs[6–9]. Other examples include the Monarch Initiative KG connects genotypic and phenotypic data across species to assist in the diagnosis of rare diseases, enabling clinicians to match patient symptoms with relevant genes even when traditional diagnostic methods fail [10,11]. Similarly, OpenTarget’s KG prioritizes drug targets by synthesizing genetic associations, expression data, and pathway information [12]. Many methods have been proposed to link knowledge graphs with large language models (LLMs) to reduce hallucinations and improve scientific utility[13–15]. As LLMs become more widely used in biomedicine, KGs can further support them through retrieval-augmented generation (KG-RAG), helping to normalize and ground generative outputs. Early efforts, such as pairing BioGPT and SciSpaCy[16,17] with structured KG-based retrieval, demonstrated how graphs could enhance scientific question answering and mechanistic reasoning. Today, more advanced approaches, such as DRAGON-AI, OntoGPT, AMG-RAG, DR.KNOWS, and KGARevion [18–22], illustrate a new generation of agentic KG-LLM integrations. These systems extend beyond retrieval to provide reliability, provenance, reasoning transparency, and medical context. Graph-based approaches offer scalable, efficient, and cost-effective ways to generate testable hypotheses, often uncovering unexpected cross-domain relationships that would be difficult to detect manually. By integrating structured knowledge from genetics, clinical records, literature, pathways, and curated knowledge bases, knowledge graphs (KGs) enable a more comprehensive and mechanistic understanding of biology and disease [7,8,23–29].

The biomedical knowledge graph (KG) landscape has been described as a “wild west” of inconsistent standards and practices [30,31]. Many KGs are developed with bespoke methods and evaluated using metrics specific to narrow applications, which severely limits interoperability and reuse. This fragmentation leads to redundant reconstruction of many similar graphs, restricts feedback to upstream data providers, and diminishes opportunities for data harmonization and quality improvement. Reuse is further constrained by incomplete documentation, infrequent updates, and insufficient evaluation, factors that collectively undermine confidence and incentivize the creation of new KGs, often as fundable but duplicative deliverables.

Ontologies provide a semantic hierarchical structure and vocabulary for integrating data. Using ontologies in KGs allows complex relationships and links to different concepts, such as genes, traits, or phenotypes, to be represented in a standardized and computable manner [4,32]. For ontology creation, there are defined standards and guidance such as OWL, DOS-DP and ROBOT templates[33–35]. The OBO Foundry provides ontology best practice metrics such as responsiveness, maintenance, format, and others[36]. The Bioregistry is an open source curated database of prefixes and metadata for ontologies, controlled vocabularies, and other semantic types[37]. Bioportal encourages reuse of ontologies through an open source repository[38]. These resources have encouraged rigorous creation and maintenance of ontologies in order to ensure that data can be shared across projects. KGs, built from ontologies and data sources, are now experiencing similar issues as ontologies were before these standards and resources were built. While the ontologies and data sources generally maintain consistent versioning, detailed documentation, and structured formatting, these features are not often retained in the KGs assembled from these parts. It is time that we have the same level of standards for KGs.

Non-biomedical KGs can also provide important lessons in data management and graph creation. Google, Facebook, IBM and others use KGs to organize and deliver information. The size of these KGs necessitates a consistent underlying schema or structure[39]. Without a consistent underlying structure, it would be nearly impossible to create reliable graphs of these magnitudes. Standards in KG development are similarly important in industry and other scholarly domains [40,41], and although use cases vary, the principles are applicable to biological domains as well. Unlike social graphs such as Facebook’s, which are largely centered on people and their interactions, biomedical KGs must capture a far more complex landscape. In biomedicine, the same two entities can be linked by different types of relationships depending on the context, and those relationships may change as new evidence is discovered. In some cases, the relationships may even appear contradictory but remain accurate within their specific experimental or biological setting. Additionally, biomedical KGs must also integrate across multiple biological scales (from molecules to cells to organisms to populations) and capture qualifiers such as tissue, species, or regulatory direction. Combined with the need for high accuracy, since an incorrect relation could directly influence scientific or clinical decisions, this semantic diversity and multi-scale integration make their construction and maintenance uniquely challenging.

To address these limitations, a number of community efforts, prominently including contributions from the Monarch Initiative, have developed resources to enhance the accessibility, reusability, and standardization of biomedical KGs. KG-Hub provides community practices and tooling for KG discovery, harmonization, and reuse, in a manner analogous to the role of the OBO Foundry in ontology development [36]. Within this framework, the KG-Registry catalogs metadata describing graph creators, data sources, organizations, and associated code, thereby facilitating identification and reuse of existing KGs[42]. Complementary initiatives include the (Re)usable Data Project, which evaluates and clarifies licensing to promote responsible distribution of biomedical data—an important consideration given that KGs typically integrate sources with heterogeneous reuse conditions[43]. The Biolink Model is a standardized framework for representing biological entities, their attributes, and relationships[44]. Adoption of Biolink has been critical to ensuring interoperability, and associated standards such as KGX exchange format leverage Biolink to enable subgraph extraction and integration across resources. Broader community efforts, including the NCATS Biomedical Data Translator Consortium[45], and Translator KGs such as RTX-KG2, ROBOKOP, Clinical KG, and SPOKE[1,46–48], demonstrate how strong, schema-driven approaches based on shared formats and registries provide the foundation for disseminating standardized and reusable KGs.

Versioning is critical for the reliability and reuse of KGs. Lessons from ontologies illustrate this need: a longitudinal study of the Gene Ontology (GO) showed that annotation changes over a decade produced divergent interpretations of biological experiments, with enrichment results differing substantially between earlier and later GO releases[49]. Such discrepancies arose from biases including annotation and literature bias, underscoring the dynamic nature of biological data and the necessity of regular updates. Outdated KGs risk propagating obsolete or misleading results, particularly when based on stale source data. To support reproducibility, it is desirable for past KG versions to remain accessible for comparison with newer iterations. However, practical barriers—most notably the large size of KGs and the resource demands of hosting and bandwidth—make comprehensive version archiving difficult.

Guidance can be drawn from standards used in other data domains. The FAIR, TRUST, and O3 principles provide frameworks for sustainable, interoperable data resources[50–52]^,^. FAIR is Findability, Accessibility, Interoperability, and Reusability, and its recommendations include use of controlled vocabularies for metadata, schematic interoperability across sources, and modular design to enhance flexibility. Vogt et al. (2024) argue that modularity-oriented approaches to KG construction improve compatibility, findability, and reproducibility[50]. TRUST principles extend FAIR by emphasizing long-term stewardship of digital repositories, while O3 highlights sustainability through open data, code, and infrastructure. Yet, none of these frameworks fully address the unique requirements of KG assembly and reuse. For this reason, we defined criteria tailored to KGs and applied them to a set of prominent, publicly available graphs.

Several biomedical KG reviews have focused on KG use cases and methods; but not their creation and reusability [6,53–55]. We were inspired by *Bonner et. al* [54], where they examined schema, relations and data mappings, and licensure for six drug discovery KGs, but not KG evaluation and examples on their intended purpose. They concluded that three main things missing from these KGs are sufficient documentation, regular updates, and consistent dataset versioning. In response, we present a systematic approach and set of KG metrics designed to improve interoperability, rigor, reuse, and reproducibility. By addressing these foundational issues, we aim to foster more collaborative and modular development across the community and to enable researchers to focus on applying KGs to drive new discoveries and achieve real-world impact.

## Methods

Here, we review a subset of 16 biomedical KGs[1,7–10,23,46–48,56–62] with a focus on reusability, data standards and openness. All metrics are evaluated through the lens of “*is it publicly accessible/true?”* Features only accessible through subscription or membership in a specific group are not counted. The features were assessed as a strictly *“yes”* or *“no”* system; partial compliance counted as a full *“yes.”* Only features that were completely missing or could not be found were counted as a *“no.”* Due to the variation in how KGs define, represent and approach concepts we kept our criteria intentionally broad to avoid penalizing approaches following different standards. Our goal was to give credit for efforts towards implementing or adhering to standards.

KGs were evaluated manually by domain-expert curators addressing specific subcriteria for each Principle (see below). For each KG, one of a team of six reviewers retrieved the most recently available version of the KG’s website as well as the contents of any associated software repositories (e.g., from GitHub) and scholarly publications. The review was then secondarily reviewed by another team member. A critical review of KG code and files is outside the scope of this work and was not performed (e.g., we did not determine how much time was required to build any KG). As with the OBO Dashboard and the ReusableData initiatives[36,43] 24,33, it is our hope that this review process will encourage KG stewards to improve their documentation and promote reuse and interoperability.

### Principles

We developed specific evaluation criteria to assess the accessibility, reusability, and standardization of publicly-accessible KGs. The primary goal of these principles is to determine if one can easily access and gain specific information about a given KG so as to determine its utility for reuse. Our criteria are arranged according to six principles:

- Access Level and Type
- Provenance of Nodes and Edges
- Documented Standards, Schema, and Construction
- Update Frequency and Versioning
- Evaluation by Metrics and Fitness for Purpose
- Licensing

#### A. Access Level and Type

We evaluated each KG’s ability to be accessed easily and without barriers by members of the greater scientific community. We considered direct access to the KG and its code as well as adjacent interfaces (e.g., a REST API) and derived products (e.g., embeddings, paths). The subcriteria are:

A.1 Additional KG product accessible such as embeddings or paths
A.2 API, Neo4j or other online hosted graph database accessible
A.3 Multiple ways to download or access
A.4 Source code for making the KG is publicly accessible
A.5 KG is downloadable in its entirety

#### B. Provenance of Nodes and Edges

KG sources can vary noticeably in structure and information content. Having access to original data sources can help users understand the importance of different edges and nodes, increasing biological plausibility and classification for drug repurposing tasks[63]. Knowledge of data sources can also inform choices about a KG’s reusability: some data sources may be more or less stringent than a user may prefer, so documentation of the process from source to KG can help users to evaluate the graph’s suitability for new purposes. The subcriteria are:

B.1 List of sources provided
B.2 Sources have versions/dates/file names used
B.3 Import dependencies declared
B.4 Nodes and edges have source information
B.5 Duplicate edge management taken into consideration
B.6 Provenance of edge creation
B.7 Schema for how edge types are created; where are they coming from

#### C. Documented Standards, Schema, and Construction

KGs gain much of their value from being reusable across different applications and research contexts. For this to happen, the KG’s identifiers need to be resolvable in the greater biomedical domain. In addition, how the graph is created may change the way a user employs it. Here, we assess how the documentation of the KG best supports this. The subcriteria are:

C.1 Entities and nodes are human readable and usable in biological applications outside of KG
C.2 Identifiers support entity resolution (external identity links such as Ensembl IDs[64])
C.3 Documentation on construction is provided
C.4 Documented data transforms (for example, removed general terms from ontologies)
C.5 Uses a documented schema for construction

#### D. Update Frequency and Versioning

As KGs are created from current “known” knowledge, they risk missing new information if not updated regularly; timely, well-documented updates are necessary. This is analogous to software versioning in that knowledge of the version used in a given process encourages reproducibility and more informative evaluation of the resource’s reuse in new applications. The subcriteria are:

D.1 Clearly identified stable versions
D.2 Public tracker for requests
D.3 Contact indicated for the KG; either organization or person
D.4 Updated more than once per year
D.5 Prior versions accessible with clearly documented changes

#### E. Evaluation by Metrics and Fitness for Purpose

Many KGs claim they will help scientists in the broader biological community in a spectrum of use cases. Formal evaluations, detailed examples, and quantifiable measures of confidence are necessary to establish the community’s trust in the KG’s value and applicability to its intended use cases. The subcriteria are:

E.1 Case study/example of use provided
E.2 KG utility evaluated against other methods (can include other KGs)
E.3 Clearly defined scope
E.4 Multiple types of evaluation methods
E.5 One or more ways of measuring accuracy/confidence

#### F. Licensing

Knowledge Graph data licensing is important because it clearly defines what users are legally allowed to do with the data, such as reuse, redistribution, or modification. Without proper licensing, even openly available data may not be legally reusable, creating barriers to collaboration and scientific innovation. Clear licenses enable interoperability, sharing, and the creation of tools and resources that advance research and improve human health.

F.1 KG licensing is clearly stated and unambiguous

## Results

### KG Evaluation

**Figure 1.**
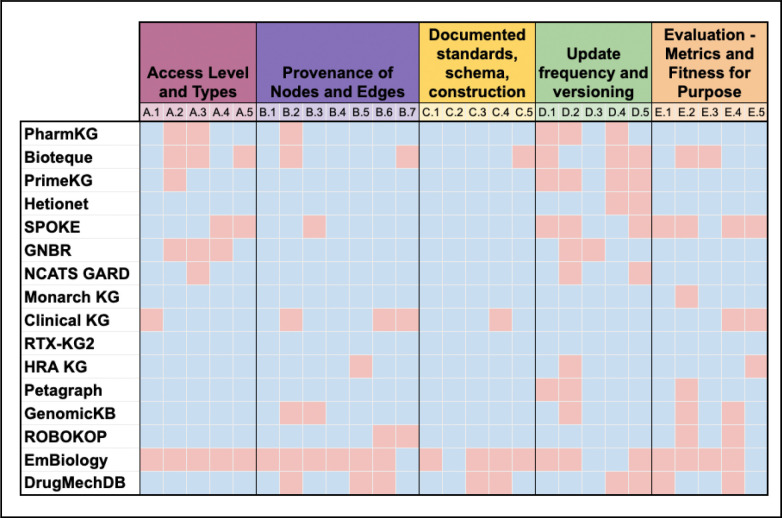
The left side lists the KGs reviewed.The top indicates the different criteria by category with each color referring to a different category. The criteria are numbered A.1, B.1,…. according to the numbering above. Blue squares indicate the KG met this principle, red indicates it did not. Licensing is not shown as it is not a binary category. Find a detailed breakdown of why each KG was assigned blue/red in the Supplemental Table Detailed Principle Analysis tab.

The sixteen chosen KGs are shown below in Figure 1 along with how they did on the criteria from above. We chose biomedical KGs of varying purposes, sizes, and creators, including those that have been widely cited such as Hetionet and Bioteque and those already in our KG-Registry. The reviewers for the Monarch Initiative’s KG, were experts outside of the Monarch Initiative in order to reduce bias.

Of note, KGs such as EmBiology and SPOKE are largely accessible only through a paywall, which has resulted in lower scores. RTX-KG2 was the only knowledge graph that scored yes on every criteria.

**Figure 2.**
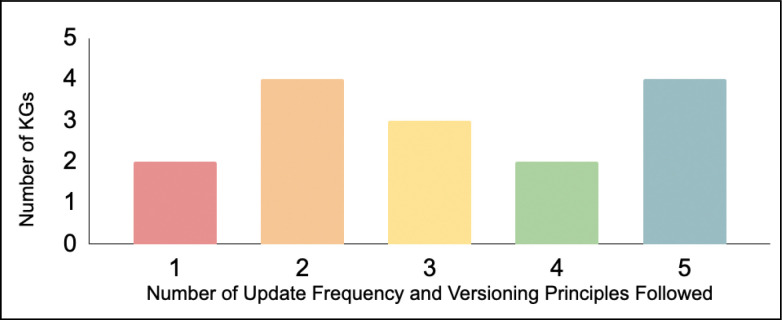
The x-axis shows the number of update frequency and versioning principles that a KG followed out of a five. The y-axis shows the number of KGs that we reviewed for each criteria in category D.

Figure 2 shows how many KGs follow principles of update frequency and versioning. Only Monarch KG, Clinical KG, and RTX-KG2 scored yes on all update frequency and version principles. All KGs except for GNBR have a contact person or organization indicated. The principles which were followed the least are having a public tracker for requests (7/16) followed by clearly identified stable versions and having prior versions accessible with clearly documented changes (9/16). This creates a lack of transparency in how KGs are maintained and improved, and contributes to problems with reproducible workflows.

### KG Nodes Types

**Figure 3.**
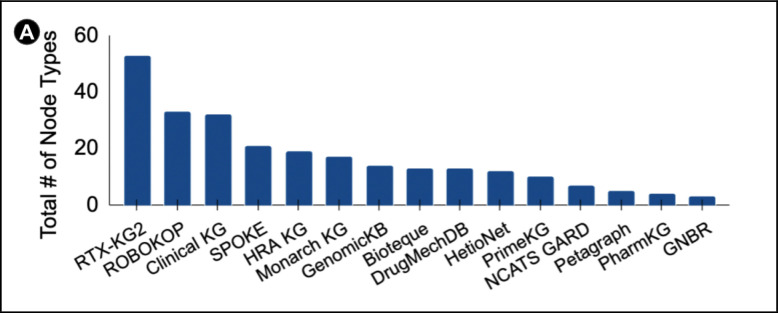

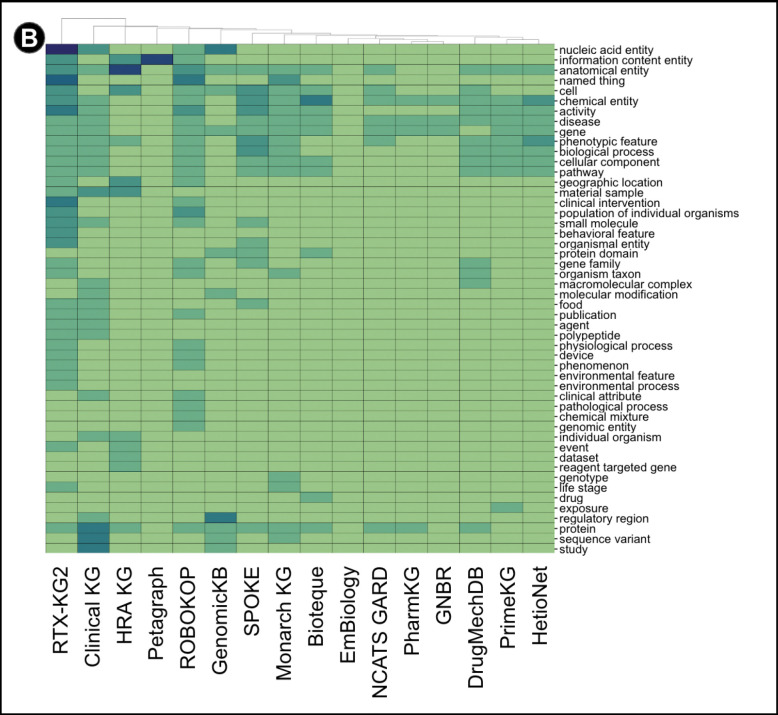
**(A)** X-axis shows the KG, y-axis shows the number of node types reported by each respective KG. EmBiology is not included in this figure as their node types are not publicly available. See Supplemental Table Node Types tab for more info. **(B)** Heatmap showing the KGs and their node types after BioLink mapping. The columns represent the KGs, the rows represent the BioLink node types. Darker blue indicates KG has more of the specific node type while green indicates the KG does not have the node type. Dendrogram across the top shows which KGs are more similar based on binomial clustering.

From the 16 KGs we reviewed the number of node types included ranged from many (52) to very few (3) with many KGs falling somewhere in between (Figure 3). Common node types were chemicals/drugs, genes and diseases. Some KGs had more custom node types such as HRA KG which used digital objects (DOs) instead of standard nodes. This design enables HRA KG to incorporate experimental data, research settings, and even 3D spatial references, giving it a distinct focus compared with KGs that limit themselves to curated biomedical entities such as genes, proteins, and diseases, without experimental data. In the case of EmBiology, publicly available information is limited: based on their website we infer that their KG includes genes, proteins, and diseases, but the details remain inaccessible because of a paywall. To better compare what kinds of nodes are used across KGs, we leveraged ChatGPT to map each node label supplied by the different KGs to the Biolink Model’s node types. The full list is provided in the Supplemental Table Node Types tab. Figure 3B shows how the different KGs cluster, according to seaborn clustermap, when node types have been mapped to BioLink. For example, the node type *“Tissues”,* present in GenomicKB, Bioteque and NCATS GARD was mapped to *“anatomical entity”*, these KGs did not have node type *“anatomical entity”* in their original list. This mapping allows us to compare KG content as RTX-KG2, ROBOKOP and Monarch all had *“anatomical entity”.*

### KG Sources

**Figure 4.**
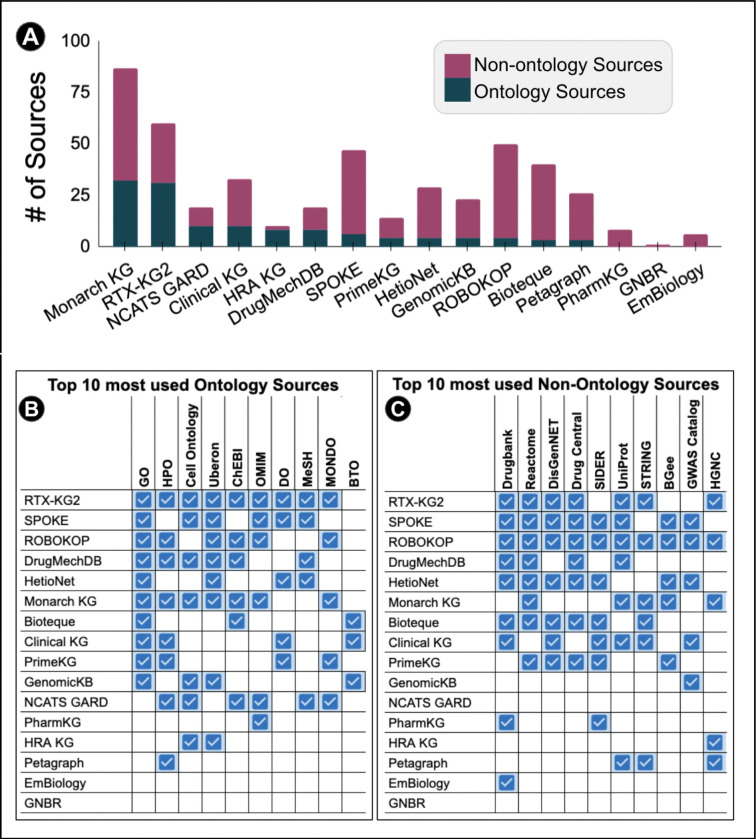
**(A)** x-axis shows the KG, y-axis shows the corresponding number of sources the KG ingests. Sources have been divided into two categories: ontologies (in purple) and non-ontology data sources (in blue). **(B)** The right side of the table shows the different KGs, the top indicates the top 10 most ingested ontology sources. A blue check indicated the KG ingested the source. **(C)** The right side of the table shows the different KGs, the top indicates the top 10 most ingested non ontology sources. A blue check indicated the KG ingested the source. More information about the KG Sources can be found in the Supplemental Table Sources tab.

Just as the number of node types varied, the number and diversity of sources also differed widely across KGs, encompassing both primary sources (original databases or ontologies) and aggregated sources (resources that themselves integrate multiple databases). As most KGs did not differentiate between primary and aggregated sources they are counted equally. Of the KGs that we evaluated (Figure 4), Monarch ingested the most number of sources at 87, with the next highest being RTX-KG2, ROBOKOP and SPOKE with 63, 50 and 47, respectively. PharmKG and GNBR had the fewest sources with seven and one. Due to EmBiology’s paywall it is unclear exactly how many sources they ingest, although we can deduce there are at least six. GNBR is a literature based knowledge graph, although constructed from many sources (papers) we classify it as having one source “other”. Some KGs included experimental data or text mined data not found in a database, which we classified under the source type “other.”. KGs that included “other” source data include ROBOKOP, Bioteque, DrugMechDB, EmBiology, and GNBR.

## Discussion

When a researcher is deciding to use a knowledge graph for an analysis or experiment, they face a decision: to create a custom graph tailored to their purpose or use an existing resource and try to adapt it to fit their use case. There are advantages and disadvantages to each approach; however, given the rapidly growing number of biomedical KGs and lack of apparent reuse, it appears that there is a tendency to create one’s own. There are several reasons for this, despite the high overlap in content and sources across existing graphs. First, it is often considered easier to make something from scratch and fully understand how it works and ensure compatibility with existing or planned workflows. This need for understanding is why documentation on KG construction is so important - and based on our evaluation, often not sufficient - to convince users to trust the creators enough to reuse. A commonly encountered challenge is that the necessary information is not consolidated or easy to find. When assessing a KG and its principles, the reviewers often had to look in multiple places such as publications, websites, and github repositories to find the relevant information. Even if the KG scored ‘yes’ on a principle does not mean it was easily scored. Second, creating novel KGs is more compatible with the way research is funded and rewarded. It may be more impressive to say “we made a thing” than it is to say “we adapted this existing data and framework”, unless there is prior evidence that reuse has a measurable benefit, research tends to follow funding priorities that incentivize creation rather than reuse or validation. As such, there has been a steady stream of similar KGs being built. While this expansion might seem productive, it often comes at the expense of exploring novel methods that could make better use of existing KGs or evaluation of applying existing KGs to novel areas of exploration. The result is a cycle of effort duplication rather than innovation. If the field eventually turns away from KGs due to limited meaningful results, the real loss will be the missed opportunity to advance methods and applications that could have demonstrated their value. By continually rebuilding what we already have, we risk exhausting interest before KGs reach their full potential.

As a community, we are not generating the evidence needed that reuse actually helps advance the field because of the lack of trust in any given KG. This paper aims to help build a set of criteria to evaluate the trustworthiness of KGs in order to help researchers better build upon each other’s work. Each KG was assessed against our criteria with a simple “yes/no” scoring system, where the number of “yes” answers provides an overall score of transparency and reusability. Well documented code and explicit descriptions of the data structure encourages methodological innovation by computational biologists. Additionally, being able to contact the authors to ask for clarity or suggestions offers another layer of trust. A good way to build trust would be for KGs to have a machine readable overview file dictating things about the KG such as date updated, authors, size, sources, etc. In this study, we do not set a strict cutoff for what constitutes a “trusted” KG (e.g., requiring a minimum number of “yes” scores), but we propose that higher scores across these criteria reflect greater trustworthiness. We also report scores across the criteria in Figure 1 to enable head-to-head comparison.

Even if users do trust existing KGs, the next question is which one do they pick? Not all KGs are created equal for all biomedical tasks. In order to confidently choose a KG or set of them, researchers need to know the different data types in the graph and the data sources used to build it. As shown in Figure 3 and 4, KGs have a varying number of node types and sources ingested. When an outside user looks to repurpose an existing KG, it needs to be clear which information is already represented and where it came from. Data transparency enables a user to take an existing KG and add or remove their own data sources in a compatible way, making it easier to repurpose. Figure 4 shows that many KGs ingest similar sources. However, this does not guarantee that the content is of equal quality once it is aggregated into a KG. An equally important aspect of KG trustworthiness is the accuracy of resource ingestion. Even when two KGs draw from the same source, they may ingest and represent the dataset in different ways. For instance, while both ROBOKOP and Monarch KG ingest some of the same sources, they often pull from different tables within those sources, leading to differences in coverage and emphasis. In another instance, PharmKG focuses on diseases above the fifth hierarchical level in MeSH while Monarch KG makes use of all diseases in MONDO. While cutting more general terms could be useful in specific tasks it would lose information content inherent to the source ontologies. Knowing these KG design choices, influences the choice of both KG and methods to use for a specific use case.

Embedding KGs may be a useful strategy for data that cannot be publicly available, such as sensitive clinical data. However, without excellent documentation, frequent updates, and robust evaluation and validation, embeddings as the sole shareable artifact have little practical value.. Embeddings are constrained to the version of information and embedding strategy used at the time of creation. There is no way for an outside user to add new information or generate embeddings using alternative techniques. Additionally, embeddings also lose the data providence and explainability that are inherent to well constructed KGs. We urge the community to further define best practices and approaches to sharing and validating embeddings, much in the same way that is currently done for bringing models to data[65,66].

When evaluations of biomedical KGs are provided, they vary greatly in scope, application and rigour. More general evaluations of a KG, such as agreement with other sources, can increase trust in the information contained, but do not provide insights into how the KG may be useful for specific analysis. Conversely, some evaluations only consider a specific use case, for example, insights into a specific disease (e.g., Alzheimer’s, epilepsy) or drug. This level of specificity inherently limits what the KG is likely to be used for in the future. A robust analysis that includes both general domain-agnostic evaluations and nuanced use-cases comparing a novel KG to existing methods or knowledge sources will generate more trust and opportunities for reuse.

When assessing performance of KGs on machine learning applications like drug repurposing, fairness in evaluation is critical. Using a validation set drawn from one of the KGs inevitably skews results towards that KG, since the topological structure of each KG differs. This bias is particularly pronounced in machine learning tasks such as link prediction, where the KG that supplied the validation set will closely resemble the topology of the known connected pairs. The other KG, by contrast, may appear to underperform simply because its structure reflects different sources and ingestion protocols. To mitigate this bias and enable more meaningful comparisons, external validation sets curated independently of either KG should be used.

Many of the KGs have overlaps in sources and node types, as expected. However, there is no guarantee that the node type in *KG (a)* is the same as node type in *KG(b)* even if they have the same name. Conversely, node type ‘a’ in *KG(a)* may have a different name than node type ‘b’ in *KG(b)* but be essentially the same data. The same source can also be ingested differently making two seemingly similar graphs more different than one would expect. Two KGs can ingest the same data but from two different places such as a primary data source (the original ontology) vs an aggregated source that has many ontologies. For example, one can ingest the primary source such as the Basic Formal Ontology (BFO) as opposed to OBO Foundry which ingests many ontologies including BFO. Primary vs aggregated source reporting could make two KGs look different on the surface when in fact they are quite similar. As KGs follow different standards and data models, there is not a good way to compare the sources and nodes in different KGs in a robust non biased way. Currently it is nontrivial to compare different knowledge graphs however, if all KGs followed similar construction methods and were in the same format such as KGX with the same metadata stored in machine readable files, comparison would not only be easier, but standard.

## Conclusion

In conclusion, for KGs to reach their full potential in the biomedical landscape we must shift from the fragmented landscape of reinvention and come together as a community towards collective stewardship. Strengthening existing KGs through rigorous documentation, transparent standards, and shared evaluation frameworks will make reuse not just possible, but preferable to building yet another graph with overlapping content. By embracing common principles of construction and machine-readable metadata, researchers will be empowered to compare, adapt, and extend KGs with confidence. The result will not only be greater trust in individual KGs, but also a fertile ecosystem where innovation flourishes because energy is spent advancing methods and applications, rather than duplicating what already exists. Only by uniting around shared standards can we ensure that KGs mature into reliable, reusable, and indispensable infrastructure for the biomedical sciences.

## Supplementary Material

Supplement 1
