# Supplementary material for: Improving Biomedical Knowledge Graph Quality: A Community Approach
Source: ArXiv. 2025 Aug 29:arXiv:2508.21774v1. Preprint. [Version 1] (PMC12407614)
Supplement: Supplement 1 [file NIHPP2508.21774v1-supplement-1.pdf]

Detailed Principle Analysis

	A. Access Level and Types				
	A.1	A.2	A.3	A.4	A.5
	Can Access something that isnt original KG (embedding, paths)	Can access by API or Neo4j or other online hosted graph database	Multiple ways to download/ access	Can access source code for making KG	Can download KG
PharmKG	Yes - PharmKG provides embeddings, learned low-dimensional representations for entities and relations via KGE models. It also provides top predicted paths for mechanistic interpretation (Fig 6) and t-SNE visualizations showing semantic structure (Fig 5).	N	N	Yes - The full source code for KG construction, processing, and the Heterogeneous Graph Attention Network (HRGAT) embedding model is provided on the GitHub repository https://github.com/MindRank-Biotech/PharmKG/tree/master	Yes - The benchmark KG is downloadable. They also provide both the clean version (final benchmark) and the raw version (with more entities and relations); the KG dataset is guarded by Google Drive permissions to access it
Bioteque	Y (Bioteque provides node-type-specific embeddings for 11 of the node types in its graph, along with downloadable sets of those individual sets of nodes mapped to their representations in the embeddings.)	N	N	Y (Source code for assembling the KG is provided in a GitHub repository, https://github.com/sbnb-irb/bioteque. This includes source-specific scripts for retrieving data components.)	N
PrimeKG	Yes - ClinicalBERT-based embeddings were used to group disease nodes, providing an embedding-derived version of the graph	N	Yes - Available via Harvard Dataverse with raw KG (kg raw.csv) and largest connected component (kg giant.csv) https://dataverse.harvard.edu/dataset.xhtml?persistentId=doi:10.7910/DVN/IXA7BM	Yes - Full source code is available on GitHub https://github.com/mims-harvard/PrimeKG	Yes - Harvard Dataverse Repo hosts the downloadable KG and intermediate files https://dataverse.harvard.edu/dataset.xhtml?persistentId=doi:10.7910/DVN/IXA7BM
HetioNet	Yes - Can access paths, DWPCs, prediction probabilities, network support breakdowns for compound–disease pairs (via Neo4j Browser & guides)	Yes - fully hosted on a public Neo4j instance with Cypher queries, guides, tutorials https://neo4j.het.io/browser/	Yes - Downloadable as JSON, Neo4j DB, TSV; also query online in Neo4j Browser; source code & intermediate datasets on GitHub, Zenodo, Figshare	Yes, the source code and scripts are public on hetio and GitHub linked in the paper https://github.com/elifesciences-publications/hetionet	Yes - Multiple export formats (JSON, Neo4j dump, TSV)
SPOKE	yes - neighborhood explorer; limited to 10k nodes; API https://spoke.rbvi.ucsf.edu/swagger/	yes - neighborhood explorer; limited to 10k nodes	yes	no	no
GNBR	yes can access themes which relate to the path types and their score	no	no	no but can get code to process data	yes but its very weirdly done; separated into files by edge types
NCATS Gard	Yes – The KG enables extraction of disease mappings, disease profiles, and pathogenesis paths, as shown in the case studies (e.g., 3-hop paths from Wilson disease)	Yes – Public Neo4j instance available: https://disease.ncats.io	N	Partially – The in-house framework stitcher is mentioned and linked (https://github.com/ncats/stitcher), but full KG generation scripts for GARD KG are not public	Y (networkx pickle)
Monarch KG	Yes, can access the mapping schema biolink model which provides sematics mappings and ontological relationships, can access downloadable graph dumps in SciGraph or KGX formats.	Yes, can access by Monarch's own webpage, API, Neo4j, downloadable Graph.	Yes, Monarch provides multiple ways to download the data such as KGX TSV, KGX JSON lines, Neo4j Dump, etc.	Yes, can access the source code for making the KG on Github.	Yes, KGX TSV, KGX JSON lines, Neo4j Dump, etc.
clinical KG	no	yes both	yes; neo4j, docker, csv, python library	yes well documented github https://github.com/MannLabs/CKG?tab=readme-ov-file	yes many different ways
RTX-KG2	Yes, RTX-KG2 is able to provide various biomedical information through queries. It is also used as backend to support ARAX's path reasoning and path ranking (https://github.com/RTXteam/RTX) .	Yes, can access by API query, and Neo4j.	Yes, multiple ways to access including downloadable versions, API (SmartAPI), web browser user interface (seems not currently working).	Yes, Github (https://github.com/RTXteam/RTX-KG2)	Yes, downloadable versions are available on Github
HRA-KG	Yes, users can ask biological questions via queries (Basic Usage: https://cns-iu.github.io/hra-kg-supporting-information/#basic-usage, Advanced Usage: https://github.com/cns-iu/hra-kg-supporting-information/blob/main/README.md)	Yes, can access by SPARQL endpoint, HRA API, and RESTful-API.	Yes, dynamic queries through its SPARQL, RESTful-API, and UIs.	Yes, Github (https://github.com/hubmapconsortium/hra-kg)	Yes, available on Zenodo and HRA CDN
Petagraph	yes, portions of the graph that are open licensed are available from https://osf.io/6jtc9/files/osfstorage	yes, (ish), neo4j dump can be downloaded if you have a UMLS license key, and there are instructions to build a neo4j from sources in the github repository. Ish, because it's not specifically hosted, you need to host it yourself.	no, (ish) completed graph can only be downloaded as neo4j, components to build the graph can also be downloaded, but that doesn't quite feel like it makes a yes	yes, ETL of upstream sources lives in https://github.com/x-atlas-consortia/ubkg-etl/	yes (with UMLS api key)
GenomicKB	Y, can submit an online query and download subgraphs that meet criteria	website with UI for creating queries, API supposely coming in v2, Neo4j dump available but not hosted by creators	Y, can use online query tool or download Neo4j dump	Y, https://github.com/tinalee-tech/KG-dataloader	Y, Neo4j dump
ROBOKOP	Y, can access subgraphs through query searches	Y: neo4j, ExEmPLAR (experimental tool for queries of Neo4j graphs), website with "question builder" feature, automat queries (cypher and TRAPI)	Y, can download or query searches in multiple ways	Y, https://github.com/RobokopU24/ORION (this is not the original GH from the paper but is now the one they are using)	Y, https://stars.renci.org/var/plater/graphs/RobokopKG/6fe13d850fdbf89c/
EmBiology	N	N	N	N	N
DrugMechDB	Y, paths that describe the mechanism of action for drug/disease indication pair	website, can view all paths or only those related to a specific drug or disease	Y Can download from zenodo or use website	Y: https://github.com/SuLab/DrugMechDB	Kind of on zenodo, but what they are calling a KG is a collection of entries for drug/disease pairs and the paths connecting them. Raw KG containing all edges between all entries does not appear to exist

	B. Provenance of Nodes and Edges						
	B.1	B.2	B.3	B.4	B.5	B.6	B.7
	List of sources provided (record sources if yes)	Sources have versions/dates/file names used	Import dependencies declared	Nodes and Edges have source information	Duplicate edge management taken into consideration	Source specifics for what triples are created	Schema for how edge types are created; where they are coming from
PharmKG	Yes — An integration of 6 sources (OMIM, DrugBank, PharmGKB, TTD, SIDER, HumanNet); plus entity features from MeSH, PubChem, BioBERT, BioGPS, and Connectivity Map	No - Do mention integrating "recent versions" of each source and give details of ID mapping and unification (e.g., Entrez Gene ID, MeSH hierarchy) but no explicit version numbers or file names for each data dump are shown in the text	Yes - The code is built on Pykeen and KG-reeval, with details for training and hyperparameters. Also mentions use of RDKit for chemical fingerprints and BioBERT for embeddings	Partially - Confirms that nodes were unified with standard IDs (Entrez, MeSH, PubChem) and that duplicate synonyms were resolved, but it doesn't state that each edge carries explicit source provenance tags — only that relations were merged and thematically assigned using Global Network of Biomedical Relationships (GNBR)	Yes - Explicitly describes merging overlapping triplets, disambiguation with synonym tables, and merging semantically similar relations using clustering	Yes - Explains how 29 relation types were derived, how Global Network of Biomedical Relationships (GNBR) semantic themes were mapped, and which sources contribute to which entity types and interactions	Yes - Documents edge semantics: "interaction," "disease-gene," "chemical-gene," "disease-chemical," and subtypes — all mapped from Global Network of Biomedical Relationships (GNBR) semantic themes and curated bases
Bioteque	Y (see https://bioteque.irbbarcelona.org/sources)	N	Y (The list of data sources is documented and includes specific mentions of source files in its data retrieval scripts, e.g., the source for COSMIC is defined here: https://github.com/sbnb-irb/bioteque/blob/master/datasets/cosmic_mutsig/get_data.sh)	Y (The sources of nodes and edges are provided in the documentation and the node files.)	Y (The 2022 Bioteque paper makes two notes about duplicate resolution: "...we first mapped the samples and genes to our reference vocabulary and collapsed the duplicates by their mean value", and "We mapped the cell lines and genes to our reference vocabularies and took the mean value whenever duplicates occurred".)	Y (The list of data sources is documented and makes specific mention of which associations are derived from which sources; see https://bioteque.irbbarcelona.org/sources)	N
PrimeKG	Yes - 20 primary data sources listed, including DisGeNET, DrugBank, UMLS, Orphanet, etc. in the paper https://www.nature.com/articles/s41597-023-01960-3	Yes - Explicit versions and download dates provided for each dataset in the Methods/Data Records section	Partially - Tools like goatools, beautifulsoup, regex scripts, and vocabulary mappings are mentioned in the GitHub repo, but not all formal dependencies are listed	Yes - Each node contains node source; edges are annotated by type and origin	Yes - Duplicates and self-loops were removed during KG preprocessing and merging	Yes - Clear documentation on what triples were derived from which resource (e.g., drug–protein from DrugBank, phenotype–disease from HPO)	Yes - The paper documented schema of 30 edge types and their origin ontologies
HetioNet	Yes - 29 sources documented; each node/edge carries source information in properties; full list with versions in paper https://elifesciences.org/articles/26726	Yes - Versions noted: e.g., DrugBank v4.2, SIDER v4.1, LINCS L1000 (Oct 2015), Pathway Commons (with date)	Yes - Input ontologies and databases fully listed with versions; also intermediate resources described (e.g., STARGEO, PharmacotherapyDB	Yes - Node/edge properties include URLs, source, license, confidence scores (for applicable edges)	Yes - Merged redundant pathways; multiple studies for same edge consolidated; non-informative gene sets removed	Yes - Explicit per metaedge: e.g., binding affinities (≤1 mM), co-occurrence p-values (MEDLINE), gene interaction specifics	Yes - Clear metagraph with 11 node types & 24 metaedges; each with documented origin & justification
SPOKE	yes in supplemental and on website	yes; pulled each week	no	yes https://spoke.rbvi.ucsf.edu/docs/index.html	yes; sort of; genes and protiens are considered different	yes; some for instance specifies PPI for humans comes from STRING; human disease ontology provides disease associated gene	yes
GNBR	yes - but NLP so not many databases used	yes - pubtator annotations from april 30, 2016	yes detailed dependancy path extraction;	yes each edge has a lot of information; NLP was used and the sentance that constructed something is listed	yes counted as a co occurance; feature not a bug used to inform network	yes - very limited	yes - NLP; had four interaction types and three node types
NCATS Gard	Y, integrates 34 distinct biomedical resources	ORPHADATA v4.0<br>Gene Ontology<br>Human Phenotype Ontology<br>GO annotations<br>Pathway Commons (PTC)<br>Pharos v3.8.0	Partially – Tools like stitcher and OWL file parsing are mentioned, but not declared formally like a software repo would	Yes – each node/edge carries provenance via stitch keys (e.g., N_Name, I_CODE)	Partially – Mappings and harmonization discussed, but no explicit deduplication process for edges described	Yes – Object properties defined per source and integration method (e.g., has_phenotype, I_CODE, N_Name)	Yes – Meta-ontology schema is described with object properties and their meanings (Table 3)
Monarch KG	Yes, 33 heterogeneous data sources including HPOA, CTD, OMIM, Orphanet, WormBase, FlyBase, MGI, dictyBase, Xenbase, SGD, RGD, PomBase, ZFIN, NCBI, HGNC, Panther, BGeeDB, Reactome, STRING.	Yes, provides the version info of the sources used: https://data.monarchinitiative.org/monarch-kg-dev/latest/index.html	Yes, declared dependencies through its build infrastructure in the monarch-ingest and kg-hub Github repo. Specifically, Monarch uses Poetry for the dependencies management.	Yes, nodes and edges contain the most upstream source and knowledge provider information.	Yes, duplcate edge and node management is taken into consideration in the construction of the KG.	Yes, the triples created are captured in multiple stages of the KG ingest pipeline including the biolink preddicate mappings and the output.	Yes, Biolink model. All relationships are mapped to Biolink predicates.
clinical KG	yes in github with links as well as supplemental table S1	no	yes in github and documents on how to download and use	yes edges have pubmed identifiers	yes; built in redundancy to assess overlap and disagreememnt of sources	no	no
RTX-KG2	Yes, 70 sources Table 1 (UMLS, SemMedDB, ChEMBL, DrugBank, Reactome, SMPDB, and 64 additional knowledge sources).	Yes, it documents the versions of the upstream sources used (https://github.com/RTXteam/RTX-KG2/blob/master/docs/kg2-versions.md).	Yes, in the requirements file.	Yes, node's ID contains source information and edge contains primary knowledge source.	Yes, it provides a pre-canonicalized graph version (RTX-KG2pre, with semantically duplicated concepts) and a canonicalized version (RTX-KG2c, withthout semantically duplicated concepts)	Yes, in the final output KG, each edge includes the source that reated that triple.	Yes, it uses Biolink Model for as the schema standard for both nodes and edges.
HRA-KG	Yes, Table 2	Yes, the type, name, and version of each DO is documented (https://lod.humanatlas.io/asct-b/).	Yes, in the hra-do-processor.	Yes, both nodes and edges have source information	Not explicit. As each digital object produces its own graph, the triples from different objects may have similar relationships, but they are stored in separate contexts.	Yes, the triples are generated by Digital Objects in in RDF format.	Yes, the KG is built based on RDF graph (CCF ontology) and standard ontologies to define relationship types between the entities.
Petagraph	yes, methods section of paper https://www.nature.com/articles/s41597-024-04070-w#Sec2	yes, ish, some source versions listed in https://github.com/TaylorResearchLab/Petagraph/tree/main/Scientific_Data_2024	yes, ubkg etl has requirements.txt, petagraph has requirements-test.txt	Yes. They either come from existing ontologies or have one file per datasource with edges and nodes that are being added.	Yes - Yes. Bidirectional edges are only for Concept–Concept; other edges are unidirectional. Redundancies are minimized using binning and source normalization	Yes, methods of paper	yes custom schema defined in paper
GenomicKB	Y, supplementary note 1	N	N	Y, tables 1 (nodes) & 2 (edges), query allows users to restrict by datasource	Considered but not de-duplicated. Users can restric querys to specific datasources and duplicate edges can be used for validation. EX: 'enhancer regulate gene' with restriction 'cell_line=GM12878' and 'data_source=EnhancerAtlas' -> 118,610 node pairs. A second query for 'variant overlap enhancer', 'enhancer regulate gene', and 'variant correlate_with gene' -> 16,871 enhancer-gene pairs from EnhancerAtlas can be validated by GTEx eQTLs	Y, table 2	Y, table 2
ROBOKOP	Y, metadata contains all source info	Y metadata includes links and versions for all sources	Y, github lists docker requirements and graph spec file with example provided	Y, node and edges contain primary knowledge source attribute and, if relationship is inferred, any algorithms/methods used	not explicitly mentioned in paper or docs, but github for creating KG/metadata records have an "edge_normalization_version" so it appears they do	"one-hop" connection schema has summary of all edge types (node 1 type, node 2 type, relationship), and individual edges include the original data source, but sources per triple type were not explicitly stated/summarized	"one-hop" connection schema has summary of all edge types, and individual edges include the original data source, but sources per edge type were not explicitly stated
EmBiology	N	N	N	N	N	N	Y, changed yearly in their baseline updated but cannot see what schema is without paid account
DrugMechDB	table with node sources. Indications: DrugCentral (according to GH, randomly sampled from availble drug-disease pairs, does not include all of them), paths: Mechanism of Action section from DrugBank, description section within Inxight Drugs, review articles, GeneOntology , UniProt, Reactome, and well-sources Wikipedia articles. No Primary experimental results are included. All are verified by curators	Date given for DrugCentral download, cannot find info for other sources	Y, there are requirements.txt and .yaml files in the KG's downloadable .zip file on Zenodo (https://zenodo.org/records/8139357) showing the dependencies.	each entry consist of 'graph', 'links', 'nodes', and 'reference' keys. Graph contains the drug/disease pair, links contains relationships informative to the indication, nodes contains information about all entities in link, and reference contains all the sources for the entire relationship but not specifically for individual nodes/edges	paths are manually curated	edges between informative nodes in a path do not contain orignal source infromation. Informative paths have citations for the entire path, not indivudal triples within them.	BioLink, but does not specify exactly what sources are used for each edge type

	C. Documented standards, schema, construction				
	C.1	C.2	C.3	C.4	C.5
	Data is human readable and usable in biological applications outside of KGG	Identifiers are resolvable for entity resolution (external identity links such as Ensembl IDs)	Documentation on construction is provided (did they make an effort?)	Documented data transforms (ex: removed general terms from ontologies	Uses a documented schema for construction
PharmKG	Yes - All entities explicitly use standard biomedical identifiers and the structure is designed to support drug repurposing, target discovery, adverse reaction prediction, etc., in real-world biomedical tasks	Yes - Genes use Entrez Gene IDs; diseases use MeSH IDs; chemicals use PubChem IDs	Yes - Explains the construction in detail, including entity filtering, merging, disambiguation, feature extraction, and the final schema design	Yes - Removed trivial entities, merged low-level symptoms into MeSH parent diseases, clustered and merged redundant relation types, applied PCA for feature reduction, and explain all steps clearly	Yes - 29 defined relation types with source mapping and entity categories; and semantics from Global Network of Biomedical Relationships (GNBR) themes and curated bases
Bioteque	Y (the provided node files are provided as TSVs, though assembly of a full KG would require running the graph assembly code. It appears that the assembly code also produces nodes and edges in a TSV format.)	Y (node identifiers are from clearly defined sources and expressed as CURIEs)	Y (Documentation regarding the assembly code is provided; see https://github.com/sbnb-irb/bioteque)	Y (each source has its own transform code and documentation, provided on the GitHub repo, https://github.com/sbnb-irb/bioteque)	N
PrimeKG	Yes - Clinical and pharmacological text features are readable and interpretable (e.g., Mayo Clinic descriptions, DrugBank pharmacodynamics)	Yes - Uses Mondo, DrugBank, HPO, MeSH, Entrez Gene IDs, and UMLS CUIs, which are mappable and resolvable via external resources	Yes - Extensive paper + GitHub repo	Yes - Transformations like self-loop removal, duplicate dropping, phenotype-disease resolution, and mapping across ontologies are documented	Yes - Node and edge formats, and their standardized schema, are explained in the methodology and data files
HetioNet	Yes - Uses standard biomedical IDs: Entrez, UMLS, MeSH, DO, Uberon	Yes - Entrez Gene, DOID, MeSH IDs, InChIKeys used for easy cross-referencing	Yes - Extensive: paper + Thinklab logs + GitHub issues + detailed guides	Yes - Explained pruning (e.g., filtering Uberon terms, merging pathways, restricting GO terms by size)	Yes - Metagraph is the explicit schema; node and edge types clearly defined
SPOKE	yes no custom data transforms that are KG specific	yes uses Ensembl	yes edge and node types specified with source	yes; data is downloaded and integrated as is	yes uses Biolink when practical
GNBR	yes all information coming from publications outside of KG	yes uses UMLS IDs for rare diseases	yes specifies where disease and drug nodes come from	yes eliminated paths containing dependencies of type conj	yes self documented and reported with downloads
NCATS Gard	Yes – Disease profiles, drug-disease associations, and harmonization rules demonstrate biomedical applicability	The paper explicitly mentions using "Unique Ingredient Identifier (UNII)," "MONDO ID," and "OMIM ID" for mappings, indicating that external identifiers are a core part of their entity resolution strategy	Yes – Detailed methods section describes data collection, mapping strategies, and harmonization	The paper notes that "data cleanup was performed," mentioning specific examples like restricting prefixes for OMIM IDs and Orphanet IDs. It also discusses the challenges of programmatic annotation, such as preventing the annotation of generic terms like "Disease" or "Syndrome"	The paper outlines a "meta-ontology" that serves as the schema for the knowledge graph. This includes a clear definition of primary classes and object properties used to structure the data; documented in tables 2–4
Monarch KG	Yes, Monarch KG is both human-readable and usable. Monarch provides its data in tablular formats that are easy to understand. Also, Monarch KG data and its Monarch initaitive webpage can be used in bioinformatics & biomedical analysis, machine learning, NLP, LLMs, visualization & exploration, etc.	Yes, supports resolvable and externally linked identifiers. Monarch KG uses curie-style IDs that follow identifier conventions and are mappable to URLs for resolution. Also, it includes a column called xref which contains a list of external identifiers.	Yes, Monarch made great effort to document its KG construction including data ingestion, transformation, schema usage, and KG merging.	Yes, Monarch documented data transforms including the excluded nodes and dangling edges.	Yes, Monarch uses a documented schema, Biolink Model, and its monarch-ingest pipeline for construction.
clinical KG	yes use all external identifiers; ontolgies	yes used standardized identifiers for nodes not described with exsiting terminoloies or ontologies	yes well documented in the paper and a github is well done	no	yes custom; defined in paper figure 3A
RTX-KG2	Yes, it is used for other biological applications such as answering translational science questions, drug repositioning, identifying new therapeutic targets, and understanding drug mechanisms.	Yes, it uses resolvable IDs for the entities.	Yes, it has clear and step by step documentation on construction on its Github repo	Yes, in Appendix	Yes, Biolink model and extract-transform-load (ETL) approach for construction.
HRA-KG	Yes, the data is human readable and usable in various biological applications.	Yes, it uses resolvable IDs for the entity resolution such as UBERON: 0013702 and ENSG00000175899.	Yes, it provides comprehensive documentation on its construction process (HRA KG Construction and Deployment).	Yes, it documents data transfroms such as data normalization and enrichment.	Yes, ASCT+B tables and Common Coordinate Framework (CCF) Ontology.
Petagraph	Yes, csv files	Yes, concept CUI with codes corresponding to common external ID/s	Yes, well documented github	guidlines for formatting ontologies in user guide, method explain preprocessing steps	Start with ontologies and standards in the UBKG and add in omics data based on their paper defined in the schema
GenomicKB	Y, they have a web interface (https://gkb.dcmb.med.umich.edu/) presenting the entities and edges in a human-readable format. It can be used to answer genomics-related questions and conduct biological analysis	Y, uses one external ID when possible and defines globaly unique IDs for entities without such as ChIP-seq peaks	Y, github and supplement	Y, sup Note 1 Data Collection and Processing	Uses custom schema not an existing one but does provide documentation
ROBOKOP	Yes, json files	Y, choose one external representative entity ID and map synonyms using existing equivalencies and specailized rules	Y, github repo for construction with reasonable documentation on how to use it in general, and metadata files include exact versions of functions that were used when creating their KG	not explicitly described but metadata contains all preprocessing steps per source with versions of each normalization strategies used	BioLink
EmBiology	N	Not explicitly stated but they use ML for entity recognition so probably	N	N, lots of this is based on NLP/text mining approaches but preprocessing of sources is not documented	N
DrugMechDB	yes, json files	yes, all nodes come from existing sources	Not really. Everything is "manually curated"	N	BioLink

	D. Update frequency and versioning				
	D.1	D.2	D.3	D.4	D.5
	Clearly Identified Stable Versions	Public tracker for requests	Is there a contact indicated for the KG either org or person	Updated more than once per year	Prior versions accessible with clearly documented changes
PharmKG	No - There is no formal semantic versioning scheme mentioned for the dataset or codebase	No - There is no mention of a public tracker for feature requests or issues	Yes - Two corresponding authors are provided with full institutional contacts (Prof. Yuedong Yang and Dr. Zhangming Niu) in the published paper (https://academic.oup.com/bib/article/22/4/bbaa344/6042240)	No - No evidence yet. They state they plan future expansions (e.g., transcriptomics, clinical data, auto-extraction) but no recurring updates are published so far	Partially - They do provide the raw version alongside the final benchmark on GitHub but do not maintain a changelog across multiple yearly versions
Bioteque	N	Y (the GitHub repository at https://github.com/sbnb-irb/bioteque is public and permits issue creation).	Y (it's never explicitly stated as a contact, but the responsible organization, the Structural Bioinformatics and Network Biology Group at the Institute for Research in Biomedicine Barcelona, is identified along with a link to their home page)	N	N
PrimeKG	No - No version tags (e.g., v1.0, v1.1) are mentioned or used on Dataverse or GitHub	No - GitHub Issues tab is not actively used for public feature requests or bug tracking	Yes - Maintained by Zitnik Lab at Harvard with lab contact and GitHub maintainers listed	No - Only one release version is available as of now (May 2022)	No - No archived prior versions or changelog indicating updates
HetioNet	Partial - "v1.0" labeled, but no formal version history beyond initial	Partial - Thinklab (now static); issues can be filed on GitHub	Yes - Daniel Himmelstein and team, contactable via GitHub, Thinklab archives, paper	No - Only v1.0 publicly released so far	No - Early versions mentioned but no archived download versions listed
SPOKE	no	no	yes email provided	yes updated weekly according to paper	no
GNBR	yes 7 versions available	no	no	yes but not updated since 2019	yes and indicated which version was used in the paper
NCATS Gard	No	No	Yes – Corresponding author: qian.zhu@nih.gov and NCATS/ NIH team affiliation listed	Yes - Suggests updates over time; indicates continually updated	No
Monarch KG	Yes	Yes, Monarch provides public tracker for feature requests, bug reports on it Github repo.	Yes, the contact is Monarch Initiative. Contact information is provided.	Yes, the KG is updated every month.	Yes, prior versions are accessible. And there is a dashoboard for QC showing what the changes are between any two versions. https://qc.monarchinitiative.org/#monarch?dataset=Development&kgVersion=2025-07-09
clinical KG	yes three versions	yes; encouraged through github	yes mann labs	yes; newest is from 2022	yes
RTX-KG2	Yes, it is using semantic versioning (e.g., KG2.7.3)	Yes, provides public tracker for requests, bug reports on it Github repo.	Yes, it provides contact information of the KG2 Team.	Yes, once per month (mentioned in Discussion).	Yes, the prior versions are accessible (https://github.com/ncats/translator-lfs-artifacts/blob/main/README.md) with documented changes (https://github.com/RTXteam/RTX-KG2/blob/master/docs/kg2-versions.md).
HRA-KG	Yes, e.g., v2.3.	Not explicitly mentioned, but there are multiple Github Issues portals for users to post requests, reports bugs, and discussions.	Yes, contact information is available to reach out to the team (https://humanatlas.io/about#editorial-board)	Yes, about 2 time a year (https://github.com/hubmapconsortium/hra-kg/releases).	Yes, prior versions are accessible on Github (https://github.com/hubmapconsortium/hra-kg/releases). Changed are documented (https://humanatlas.io/release-notes/v2.3).
Petagraph	No, lists date last updated but no version	No	Yes, contributors listed https://osf.io/6jtc9/	Yes, frequent small updates	recent acitivty documents date of all changes and person who made them but prior versions not accessible https://osf.io/6jtc9/
GenomicKB	Y but still on version 1.0 despite multiple upates listed	N, FAQ says to email the team	Y, website has a contacts button with name/emal for both technical details and accessing the data/web server	During 2022-2023 yes but not since then	Clearly documented chages with dates but prior versions not available. *Paper states that they store all versions internally and can revert if unexpected changes are ever made to their graph but these are not accessible publicaly
ROBOKOP	Y, https://stars.renci.org/var/plater/graphs/RobokopKG/6fe13d850fdbf89c/RobokopKG.meta.json	Y, github	Contact form provided on website and weekly office hours over zoom	Yes - ROBOKOP was built 6 times in 2024 and 3 times so far this year. - as in this metadata file - https://stars.renci.org/var/plater/graphs/RobokopKG/6fe13d850fdbf89c/RobokopKG.meta.json	accessible for bulk download. No chnage log, but each comes with metadata that documents all source ingests (including versions) and preprocessing steps used for each source
EmBiology	N	N	Contact form (for sales team)	Y, cannot confirm but website states yearly update of terminology/rules, quarterly update of clinical trial data, weekly update of relationships extracted from publications	N
DrugMechDB	Y	GitHub Issues. Also includes a guide for how users can submit their own curated paths.	indirectly. website indicated the lab who owns it, but does not provide a point of contact	N (V1 released in 2019 with one update less than a year later, same with V2 in 2023, no other updates)	All versions availble, V2 has publication associated but detailed change logs for minor updates not available

	E. Evaluation - Metrics and Fitness for Purpose					F. License Information
	E.1	E.2	E.3	E.4	E.5	
	Do they have a case study/example of use	KG utility evaluated against other methods (can include other KGs)	Clearly defined scope	Multiple different evaluation methods	One or more ways of measuring accuracy/ confidence	
PharmKG	Yes - Provide detailed case studies for Alzheimer's disease and Parkinson's disease for drug repurposing and target discovery, with literature validation, top scored predictions, and visualized paths	Yes - Benchmarked Heterogeneous Graph Attention Network (HRGAT) and 9 other KGE baselines on Hetionet and PharmKG side by side and analyzed results	Yes - The paper is a dedicated benchmark for evaluating KGE models in biomedical relation prediction, with explicit focus on drug repurposing, target identification, and multi-relation prediction tasks	Yes - Link prediction (MRR, Hits@k), downstream tasks (AUROC, AUPRC), t-tests for significance	Yes - Multiple: MRR, Hits@1/3/10/100, AUROC, AUPRC, p-values for statistical tests, plus visualization and interpretability checks with t-SNE plots and path analyses	No explicit license (restricted use). The paper notes: "© The Author(s) 2020. Published by Oxford University Press. All rights reserved." But does not reveal license information (nor does the GitHub repo)
Bioteque	Y (Examples of use are described in the 2022 Nat Comm paper; an example of generating predictions for drug repurposing is provided.)	N	N	Y (Multiple evaluation methods are provided in the 2022 Nat Comm paper, primarily for embedding evaluation.)	Y (Multiple validation methods are provided in the 2022 Nat Comm paper, including two distinct analyses involving gene expression data and protein-protein interactions, respectively.)	CC BY 4.0
PrimeKG	Yes - Autism case study demonstrates disease concept resolution and clinical alignment	Yes - Compared to other KGs (e.g., SPOKE); benchmarks and references to prior systems included	Yes - Focused on disease-centric precision medicine with defined coverage: 17,080 diseases, 10 biological scales, 20 sources	Yes – Structure connectivity, edge density, text embedding-based grouping, and clinical relevance tested	Partially - Uses similarity thresholds (e.g., cosine ≥ 0.98 for disease grouping); no formal metrics like precision/recall provided	CC BY 4.0
HetioNet	Yes - Nicotine dependence (bupropion), epilepsy predictions (acamprosate)	Yes - Compared to PREDICT, Guney et al., Cheng et al.; used baselines & permutation	Yes - Designed for systematic drug repurposing + broader knowledge integration	Yes - DWPC + AUROC + permutation + cross-validation + external test sets (DrugCentral, ClinicalTrials.gov)	Yes - Probability scores, cross-validated elastic net, path-level contribution breakdowns, AUROC	CC0 1.0
SPOKE	no	no	yes; biomedical applications such as drug reuposing, disease prediction, embedding EHR data	no	no	CC BY 4.0
GNBR	yes drug disease indications ; 2 case studies	yes; compared to gold standard drug indications; relationships from other databases for those gotten using NLP methods	yes drug repurposing	yes case studies; drug-disease path analysis; validation using DGIdb; used lit review to verify novel drug	yes performance on gold standard drug indications calculate AUROC; dendrograms of the themes	CC BY 4.0
NCATS Gard	Yes – Four detailed case studies: disease mapping, disease profiling, data harmonization, pathogenesis exploration	The paper compares its approach with other similar efforts, such as the semantic Diseasecard and the Monarch Initiative, placing its own work in the context of the broader field; but no explicit benchmarking	Yes – Focused on rare diseases, especially those curated in GARD, and integrating relevant biomedical data	Partially – Demonstrates utility via case studies but lacks formal quantitative evaluation (mapping stats in tables 6,7,10)	The paper's semi-automatic mapping process for FDA orphan designations includes a manual curation step where curators labeled mappings as "Done," "Approximate," or "Failed," providing a clear way to measure the confidence or accuracy of the mappings	CC BY 4.0
Monarch KG	Yes, Monarch KG is used in Exomiser which is a tool to annotate variants and has a ChatGPT plugin through RAG to support information search. It is also integrated into the GRAPE library for graph analysis and machine learning.	No	Yes, Monarch aims to harmonize the data across the fields (gene, disease, phenotype across species) to facilitate the discovery of disease mechanism and aid the disease diagnosis.	Yes, created Monarch Quality Control (QC) Dashboard for quality metrics that are specific to Monarch. In addition, it developed Phenotypic Inference Evaluation Framework (PhEval) to evaluate the analysis of its tool Exomiser.	Kind of but not explicit. The edge data contains columns like provided_by, publications, and has_evidence to measure the accuracy or confidence.	CC BY 4.0
clinical KG	yes; non-alcoholic fatty liver disease; cancer biomarkers; identify theraputic options for chemorefractory cases	yes compared their ability to identify cancer biomarkers with paper from 2018	yes harmonization of proteomics with other omics data	no	no	C BY-NC 4.0
RTX-KG2	Yes, it is currently being used by multiple Translator reasoning agents such as ARAX (Autonomous Relay Agent X).	Yes, it is compared to four other KGs (Hetionet, SPOKE, the SRI Reference Knowledge Graph, and ROBOKOP)	Yes, it is a part of NCATS Biomedical Data Translator project to support automated biomedical reasoning and question answering. It aims to create a semantically standardized, computable, and interoperable biomedical KG that supports translational reasoning and biomedical discovery.	Yes, it is not only evaluated with other KGs, but also evaluated on the tools that utilize it such as ARAX, mediKanren, BioThings Explorer, and ARAGORN.	Yes, the nodes and edges contain evidence, provenance, and other information for measuring accuracy and confidence.	CC BY 4.0
HRA-KG	Yes, they have several use cases and applications (Usage Notes).	No, The HRA-KG is compared with other KGs only from technical perspective, such as the number of nodes, edges, and technologies used.	Yes, HRA-KG is focused on representing the healthy human body in semantics terms including anatomical structures, cell types, and biomarker and spatial terms such as 3D reference organs.	Yes, it has hra-ols used by OLS for validation (https://lod.humanatlas.io/collection/hra-ols/latest/) and has Validation report (https://github.com/hubmapconsortium/3d-hra-ref-object-validation) (Supplementary).	Not explicit, but it has HRAlit which links the HRA DOs to publications, experts, experimental datasets (Supplementary).	CC BY 4.0
Petagraph	Yes, several in paper	No	Yes integrating/analyzing multiomics datasets	link prediction tasks auROC, precision-recall, top tissues associated w a disease, and shortest path analysis of subgraphs	Yes. AUC-ROC, Precision-Recall curves, common neighbors vs random, structural metrics compared to randomized graphs	CC BY-NC-ND 4.0
GenomicKB	The paper provides examples of specific queries that can be performed through the website that would normally require coding and integrating multiple data sources, but it is limited to knowledge extraction and does not show how this could be used to make novel biological discoveries	N	Y, coding free queries of human genomic information including both functional, strutural, epigenetic and other relationships	N	Kind of but not explicitly. Since this tool is all about data querying, confidence could be assesed by creating multiple queries like the example of using eQTLs to "validate" EnhancerAtlas relationships	CC BY-NC 4.0
ROBOKOP	Y, 4 ranging from simple linear queries to discovering mechanistic links	N	Yes - the scope is clearly defined because the paper focuses narrowly on presenting ROBOKOP KG and KGB as tools for federating biomedical sources into a usable KG, illustrating this through a few case examples. It does not try to claim to solve all KG problems, but sticks to integration via APIs and synonymization	N	Y, informative subgrahs are ranked according to number of supporting publications and literarure co-occurence of entity pairs	Open-Source MIT License
EmBiology	N	N	Not clearly stated but appears to be drug discovery for private companies	N	Confidence thresholds for relationships determined by number of supporting publications	Copyrighted to Elsevier
DrugMechDB	N	They used an external KG to evaluate agreement between types of associations present in both graphs and calculate a p-value representing when the simulated percentage of matching was greater than or equal to the observed percentage	Yes, mechanisms of action by which a drug treats a disease	N	agreement with MechRepoNet	CC0 1.0 Universal

BioLink Node-Type Mappings

Unstructured Label (Node-Type)	Biolink Class	
Activity	activity	
Agent	agent	
Analytical Sample	material sample	
Anatomy/Anatomical regions/Anatomical Structure	anatomical entity	
Anatomical Entity	anatomical entity	
Anatomy Cell Type	cell	
Antibody	protein	
Abtibody Panel	reagent targeted gene	
Behavior	behavioral feature	
Behavioral Feature	behavioral feature	
Biological Activity	activity	
Biological Entity	named thing	
Biological Processes	biological process	
Biological Sample	material sample	
Biomarker	phenotypic feature	
Cells	cell	
Cell Lines	cell	
Cell types	cell	
Cellular Components	cellular component	
Cell Summary	cell	
Chemicals / Chemical Entity/ Drugs	chemical entity	
Chemical Mixture	chemical entity	
Chromosome	nucleic acid entity	
Clinical Attribute	clinical attribute	
Clinical Intervention	clinical intervention	

BioLink Node-Type Mappings

Unstructured Label (Node-Type)	Biolink Class
Clinical Variable	clinical attribute
Clinically Relevant Variable	clinical attribute
Code	information content entity
Cohort	population of individual organisms
Collision Summary	event
Complex	macromolecular complex
Compounds	chemical entity
Concept	information content entity
Corridor	geographic location
Dataset	dataset
Definition	information content entity
Devices	device
Diseases	disease
Domains	protein domain
Donor	individual organism
Enhancer	regulatory region
Environmental Features	environmental feature
Environmental Processes	environmental process
Enzymatic Activity	activity
Epigenomic Features	molecular modification
Event	event
Exon	nucleic acid entity
Experiment	study
Exposures	exposure
Extraction Site (Spatial Entity)	anatomical entity

BioLink Node-Type Mappings

Unstructured Label (Node-Type)	Biolink Class
Food	food
FTU Illustration	information content entity
FTU Illustration Node	information content entity
Functional Regions	regulatory region
Genes	gene
Gene Family	gene family
Genomic Entity	genomic entity
Genotype	genotype
Geographical Location	geographic location
Gross Anatomical Structure	anatomical entity
GWAS Study	study
Information Content Entity	information content entity
Information Resource	information content entity
Insulator	regulatory region
Known Variant	sequence variant
Landmark	geographic location
Life Stage	life stage
Macro Molecular Complex	macromolecular complex
Material Sample	material sample
Metabolite	small molecule
MicroRNA	nucleic acid entity
Millitome Extraction Site	anatomical entity
Modification	molecular modification
Modified Protein	protein
Molecular Activity	activity

BioLink Node-Type Mappings

Unstructured Label (Node-Type)	Biolink Class
Molecular Entity	named thing
Molecular Functions	activity
Molecular Mixture	chemical mixture
Named Thing	named thing
Non Coding RNA Product	nucleic acid entity
Nucleic Acid Entity	nucleic acid entity
Nutrients	small molecule
Organisms	organismal entity
Organism Taxon	organism taxon
Organismal Entity	organismal entity
Orthology	gene family
Pathological Process	pathological process
Pathways	pathway
Peptides	polypeptide
Perturbagens	drug
Pharmacologic Classes	chemical entity
Phenomenon	phenomenon
Phenotypes/ Effects/ Phenotypic Feature	phenotypic feature
Physical Entity	named thing
Physiological Processes	physiological process
Polypeptide	polypeptide
Population of Individual Organisms	population of individual organisms
Procedure	clinical intervention
Project	study
Promoter	regulatory region

BioLink Node-Type Mappings

Unstructured Label (Node-Type)	Biolink Class
Proteins	protein
Protein Domains	protein domain
Protein Family	gene family
Protein Structure	protein
Publications	publication
Reactions	biological process
Reference Organ	anatomical entity
Reference Organ Part	anatomical entity
RNA Product	nucleic acid entity
Semantic	information content entity
Sequence Variant	sequence variant
Side Effects	phenotypic feature
Small Molecules	small molecule
Somatic Mutation	sequence variant
Subject	individual organism
Super Enhancer	regulatory region
Symptoms	phenotypic feature
Term	information content entity
Tissues	anatomical entity
Tssue Block (Sample)	material sample
Tissue Section (Sample)	material sample
Transcript	nucleic acid entity
Treatment	clinical intervention
User	agent
Variants	sequence variant

BioLink Node-Type Mappings

Unstructured Label (Node-Type)	Biolink Class
Vitamins	small molecule
3D Structures	protein domain

Node types

	Total # KG per node type	RTX-KG2	ROBOKOP	Clinical KG	SPOKE	HRA-KG	Monarch KG	GenomicKB	Bioteque	DrugMechDB	HetioNet	PrimeKG	NCATS Gard	Petagraph	PharmKG	GNBR	EmBiology*
**Total # of Node types**		53	36	34	21	19	17	14	13	13	12	10	7	5	4	3	0
**Activity**	1		✓														
**Agent**	1	✓															
**Analytical Sample**	1			✓													
**Anatomy/Anatomical regions/Anatomical Structure**	4				✓	✓					✓	✓					
**Anatomical Entity**	3	✓	✓				✓										
**Anatomy Cell Type**	1				✓												
**Antibody**	1					✓											
**Abtibody Panel**	1					✓											
**Behavior**	1	✓															
**Behavioral Feature**	1	✓															
**Biological Activity**	1	✓															
**Biological Entity**	1	✓															
**Biological Processes**	8	✓	✓	✓	✓		✓			✓	✓	✓					
**Biological Sample**	1			✓													
**Biomarker**	1					✓											
**Cells**	6	✓	✓				✓		✓	✓			✓				
**Cell Lines**	2	✓						✓									
**Cell types**	2				✓	✓											

Node types

	Total # KG per node type	RTX-KG2	ROBOKOP	Clinical KG	SPOKE	HRA-KG	Monarch KG	GenomicKB	Bioteque	DrugMechDB	HetioNet	PrimeKG	NCATS Gard	Petagraph	PharmKG	GNBR	EmBiology*
**Total # of Node types**		53	36	34	21	19	17	14	13	13	12	10	7	5	4	3	0
**Cellular Components**	9	✓	✓	✓	✓		✓		✓	✓	✓	✓					
**Cell Summary**	1					✓											
**Chemicals / Chemical Entity/ Drugs**	11	✓	✓	✓			✓		✓	✓	✓	✓	✓		✓	✓	
**Chemical Mixture**	1	✓															
**Chromosome**	1			✓													
**Clinical Attribute**	1			✓													
**Clinical Intervention**	2	✓	✓														
**Clinical Variable**	1			✓													
**Clinically Relevant Variable**	1			✓													
**Code**	1													✓			
**Cohort**	1	✓															
**Collision Summary**	2		✓			✓											
**Complex**	1			✓													
**Compounds**	3				✓				✓		✓						
**Concept**	1													✓			
**Corridor**	1					✓											
**Dataset**	1					✓											
**Definition**	1													✓			

Node types

	Total # KG per node type	RTX-KG2	ROBOKOP	Clinical KG	SPOKE	HRA-KG	Monarch KG	GenomicKB	Bioteque	DrugMechDB	HetioNet	PrimeKG	NCATS Gard	Petagraph	PharmKG	GNBR	EmBiology*	
**Total # of Node types**		53	36	34	21	19	17	14	13	13	12	10	7	5	4	3	0	
**Devices**	1	✓																
**Diseases**	12	✓	✓	✓	✓		✓		✓	✓	✓	✓	✓		✓	✓		
**Domains**	2		✓						✓									
**Donor**	1					✓												
**Enhancer**	1							✓										
**Environmental Features**	1	✓																
**Environmental Processes**	1	✓																
**Enzymatic Activity**	1				✓													
**Epigenomic Features**	1							✓										
**Event**	1	✓																
**Exon**	2	✓						✓										
**Experiment**	1			✓														
**Exposures**	1											✓						
**Extraction Site (Spatial Entity)**	1					✓												
**Food**	3	✓		✓	✓													
**FTU Illustration**	1					✓												8
**FTU Illustration Node**	1					✓												
**Functional Regions**	1			✓														

Node types

	Total # KG per node type	RTX-KG2	ROBOKOP	Clinical KG	SPOKE	HRA-KG	Monarch KG	GenomicKB	Bioteque	DrugMechDB	HetioNet	PrimeKG	NCATS Gard	Petagraph	PharmKG	GNBR	EmBiology*
**Total # of Node types**		53	36	34	21	19	17	14	13	13	12	10	7	5	4	3	0
**Genes**	11	✓		✓	✓		✓	✓	✓		✓	✓	✓		✓	✓	
**Gene Family**	3	✓	✓							✓							
**Genomic Entity**	1		✓														
**Genotype**	2		✓				✓										
**Geographical Location**	2	✓	✓														
**Gross Anatomical Structure**	2	✓								✓							
**GWAS Study**	3		✓	✓				✓									
**Information Content Entity**	2	✓	✓														
**Information Resource**	1	✓															
**Insulator**	2		✓					✓									
**Known Variant**	1			✓													
**Landmark**	1					✓											
**Life Stage**	2	✓					✓										
**Macro Molecular Complex**	1									✓							
**Material Sample**	1	✓															
**Metabolite**	1			✓													
**MicroRNA**	1	✓															
**Millitome Extraction Site**	1					✓											

Node types

	Total # KG per node type	RTX-KG2	ROBOKOP	Clinical KG	SPOKE	HRA-KG	Monarch KG	GenomicKB	Bioteque	DrugMechDB	HetioNet	PrimeKG	NCATS Gard	Petagraph	PharmKG	GNBR	EmBiology*
**Total # of Node types**		53	36	34	21	19	17	14	13	13	12	10	7	5	4	3	0
**Modification**	1			✓													
**Modified Protein**	1			✓													
**Molecular Activity**	3	✓					✓			✓							
**Molecular Entity**	2	✓					✓										
**Molecular Functions**	6		✓	✓	✓				✓		✓	✓					
**Molecular Mixture**	1		✓														
**Named Thing**	3	✓	✓				✓										
**Non Coding RNA Product**	2	✓						✓									
**Nucleic Acid Entity**	2	✓	✓														
**Nutrients**	2		✓		✓												
**Organisms**	2	✓			✓												
**Organism Taxon**	3	✓					✓			✓							
**Organismal Entity**	1	✓															
**Pathological Process**	1		✓														
**Pathways**	9	✓	✓	✓	✓		✓		✓	✓	✓	✓					
**Peptides**	1			✓													
**Perturbagens**	1								✓								
**Pharmacologic Classes**	4		✓		✓				✓		✓						

Node types

	Total # KG per node type	RTX-KG2	ROBOKOP	Clinical KG	SPOKE	HRA-KG	Monarch KG	GenomicKB	Bioteque	DrugMechDB	HetioNet	PrimeKG	NCATS Gard	Petagraph	PharmKG	GNBR	EmBiology*
**Total # of Node types**		53	36	34	21	19	17	14	13	13	12	10	7	5	4	3	0
**Phenomenon**	2	☑	☑														
**Phenotypes/ Effects/ Phenotypic Feature**	6	☑		☑			☑			☑		☑	☑				
**Physical Entity**	1	☑															
**Physiological Processes**	1	☑															
**Polypeptide**	2	☑	☑														
**Population of Individual Organisms**	2	☑	☑														
**Procedure**	2	☑	☑														
**Project**	2		☑	☑													
**Promoter**	1							☑									
**Proteins**	10	☑	☑	☑	☑		☑	☑	☑	☑			☑		☑		
**Protein Domains**	2		☑		☑												
**Protein Family**	1				☑												
**Protein Structure**	1			☑													
**Publications**	3	☑	☑	☑													
**Reactions**	1				☑												
**Reference Organ**	1					☑											
**Reference Organ Part**	1					☑											
**RNA Product**	2	☑	☑														

Node types

	Total # KG per node type	RTX-KG2	ROBOKOP	Clinical KG	SPOKE	HRA-KG	Monarch KG	GenomicKB	Bioteque	DrugMechDB	HetioNet	PrimeKG	NCATS Gard	Petagraph	PharmKG	GNBR	EmBiology*
**Total # of Node types**		53	36	34	21	19	17	14	13	13	12	10	7	5	4	3	0
**Semantic**	1													✓			
**Sequence Variant**	1						✓										
**Side Effects**	2				✓						✓						
**Small Molecules**	1	✓															
**Somatic Mutation**	1			✓													
**Subject**	1			✓													
**Super Enhancer**	0																
**Symptoms**	3		✓		✓						✓						
**Term**	1													✓			
**Tissues**	4			✓				✓	✓				✓				
**Tssue Block (Sample)**	1					✓											
**Tissue Section (Sample)**	1					✓											
**Transcript**	3	✓		✓				✓									
**Treatment**	1	✓															
**User**	1			✓													
**Variants**	2			✓				✓									
**Vitamins**	2	✓	✓														
**3D Structures**	1							✓									

KG SOURCES

	Total # KG per source	Monarch KG	RTX-KG2	ROBOKOP	Bioteque	SPOKE	GenomicKB	HetioNet	clinical KG	Petagraph	DrugMechDB	PrimeKG	NCATS Gard	HRA-KG	EmBiology	PharmKG	GNBR
Total # of Sources		87	63	50	40	47	23	29	33	26	19	14	20	10	6	7	1
Alliance of Genome Resources	1	✓															
ALZHEIMERS-UNIVERSITY-OF-TORONTO	1	✓															
AGBASE	0																
ARGKB	1			✓													
ARUK-UCL	1	✓															
Anatomical Therapeutic Chemical Classification System (ATC)	1		✓														
ASP2019	1									✓							
Basic Formal Ontology (BFO)	2	✓	✓														
Bgee	5	✓		✓		✓		✓				✓					
BHF-UCL	0																
BindingDB	3			✓		✓		✓									
BioGRID	2	✓													✓		
Biolink	3		✓	✓							✓						
Biological Spatial Ontology (BSPO)	1		✓														
Brenda Tissue Ontology (BTO)	3				✓		✓		✓								
CACAO	1	✓															
CAFA	1	✓															
Cancer Cell Line Encyclopedia (CCLE)	1				✓												
Cancer Genome Interpreter	1								✓								

KG SOURCES

	Total # KG per source	Monarch KG	RTX-KG2	ROBOKOP	Bioteque	SPOKE	GenomicKB	HetioNet	clinical KG	Petagraph	DrugMechDB	PrimeKG	NCATS Gard	HRA-KG	EmBiology	PharmKG	GNBR
Catalogue of Semantic Mutations in Cancer (COSMIC)	1				☑												
CCIDB	1			☑													
Cell Miner	1				☑												
Cell Ontology (CL)	7	☑	☑			☑	☑				☑		☑	☑			
Cellosaurus	1				☑												
Cell Line ontology (CLO)	1												☑				
ChEBI	6	☑	☑	☑	☑						☑		☑				
ChEMBL	3		☑	☑		☑											
Chemical Checker	1				☑												
CIVic	1					☑											
ClinicalTrials.gov	2					☑									☑		
ClinGen	1	☑															
ClinVar	2	☑								☑							
CLUE	1				☑												
CoexpressDB	1				☑												
Common Coordinate Framework Ontology (CCF)	1													☑			
Comparative Toxicogenomics Database	0																
Compartments	1				☑												
ComplexPortal	1	☑															
Connectivity Map (CMAP)	1									☑							

KG SOURCES

	Total # KG per source	Monarch KG	RTX-KG2	ROBOKOP	Bioteque	SPOKE	GenomicKB	HetioNet	clinical KG	Petagraph	DrugMechDB	PrimeKG	NCATS Gard	HRA-KG	EmBiology	PharmKG	GNBR
**CORUM**	2				✓				✓								
**CREEDS**	1				✓												
**CTD**	4	✓		✓	✓							✓					
**Depmap**	1				✓												
**dbSNP**	1						✓										
**dbSuper**	1						✓										
**dbVar**	1						✓										
**DDPHENO**	1	✓															
**DDANAT**	1	✓															
**DFLAT**	1	✓															
**DGIdb**	1		✓														
**DGV**	1						✓										
**DIBU**	1	✓															
**Dictyostelium discoideum anatomy (DDANAT)**	1		✓														
**DictyBase**	1	✓															
**DISEASES**	4			✓		✓		✓	✓								
**DisGenNET**	7		✓	✓	✓	✓		✓	✓			✓					
**DisPROT**	1	✓															
**DGIdb**	1								✓								
**DO**	5		✓			✓		✓	✓			✓					

KG SOURCES

	Total # KG per source	Monarch KG	RTX-KG2	ROBOKOP	Bioteque	SPOKE	GenomicKB	HetioNet	clinical KG	Petagraph	DrugMechDB	PrimeKG	NCATS Gard	HRA-KG	EmBiology	PharmKG	GNBR
DOAF	2					✓		✓									
DoRoth EA	1				✓												
DrugBank	9		✓	✓	✓	✓		✓	✓		✓				✓	✓	
DrugCentral	7		✓	✓	✓	✓		✓			✓	✓					
DrugMechDB	1			✓													
Drug repurposing Hub	1				✓												
ehrlink	2					✓		✓									
EMAPA(mouse developmental anatomy ontology)	1	✓															
ENCODE	1						✓										
ENdb	1						✓										
EnhancerAtlas	1						✓										
Entrez Gene	2					✓		✓									
Ensembl	3	✓	✓				✓										
eRAM (encyclopedia of rare disease annotations for precision medicine)	1			✓													
ESPD	1			✓													
Eukaryotic Promoter Database (EPD)	1						✓										
Evolutionary Rate Covariation	2					✓		✓									
Experimental Factor Ontology (EFO)	2		✓						✓								
FAERS	1			✓													
FANTOM5	1						✓										

KG SOURCES

	Total # KG per source	Monarch KG	RTX-KG2	ROBOKOP	Bioteque	SPOKE	GenomicKB	HetioNet	clinical KG	Petagraph	DrugMechDB	PrimeKG	NCATS Gard	HRA-KG	EmBiology	PharmKG	GNBR
**FBBT**	1	✓															
**FBCV**	1	✓															
**FBDV**	1	✓															
**FDA Orphan Drug Designations Data**	1												✓				
**FlyBase**	2	✓		✓													
**FooDB**	2					✓			✓								
**Food Ontology**	1		✓														
**Foundational Model of Anatomy (FMA)**	2		✓											✓			
**FYPO**	1	✓															
**GARD**	1												✓				
**GDB**	1	✓															
**GDSC**	2				✓	✓											
**GHR**	1												✓				
**GNBR**	0																
**GO**	10	✓	✓	✓	✓	✓	✓	✓	✓		✓	✓					
**GO-Central**	1	✓															
**GO consortium**	1	✓															
**GENCODE**	1									✓							
**GENOMICS**	1									✓							
**Genomic Epidemiology Ontology (GENEPIO)**	0																

KG SOURCES

	Total # KG per source	Monarch KG	RTX-KG2	ROBOKOP	Bioteque	SPOKE	GenomicKB	HetioNet	clinical KG	Petagraph	DrugMechDB	PrimeKG	NCATS Gard	HRA-KG	EmBiology	PharmKG	GNBR
**Gene Ontology, with external relationships (GO-Plus)**	1		✓														
**GOC-OWL**	1	✓															
**GO-NOCTUA**	1	✓															
**GlyGen**	1									✓							
**GTEx**	3			✓			✓			✓							
**Guide to PHARMACOLOGY (GtoPdb)**	1			✓													
**GWAS Catalog**	5			✓		✓	✓	✓	✓								
**Harmonizome**	1				✓												
**Health Level Seven Version 3.0 (HL7V3.0)**	1		✓														
**Hetio-dag**	3			✓		✓		✓									
**Hetionet**	1				✓												
**Healthcare Common Procedure Coding System (HCPCS)**	1		✓														
**HPOMP**	0																
**Homo Sapiens Chromosomal Location Ontology (HSCLO38)**	1									✓							
**HPO**	8	✓	✓	✓					✓	✓	✓	✓	✓				
**HuBMAP Research Attributes Value Set (HRAVS)**	2									✓				✓			
**Human Developmental Anatomy, abstract**	1		✓														
**Human gene nomenclature committee (HGNC)**	5	✓	✓	✓						✓				✓			
**HGNC-UCL**	1	✓															
**Human Interactome Database**	2					✓		✓									

KG SOURCES

	Total # KG per source	Monarch KG	RTX-KG2	ROBOKOP	Bioteque	SPOKE	GenomicKB	HetioNet	clinical KG	Petagraph	DrugMechDB	PrimeKG	NCATS Gard	HRA-KG	EmBiology	PharmKG	GNBR
**Human metabolite Database (HMDB)**	3		✓	✓					✓								
**Human-to-mouse ortholog mappings (HCOP)**	1									✓							
**Human-to-mouse phenotype mappings (HPOMP)**	1									✓							
**HSAPDV**	1	✓															
**Human-to-rat ENSEMBL mappings (RATHCOP)**	1						✓										
**HumanNet**	1															✓	
**Human Protien Atlas**	4	✓			✓	✓			✓								
**HuRI**	1				✓												
**ICD-10 procedure coding system (ICD10PCS)**	1		✓														
**ICD-9, clinical modification (ICD9CM)**	1		✓														
**Incomplete Interactome**	2					✓		✓									
**Inxight Drugs**	2										✓		✓				
**IntAct**	4		✓	✓	✓				✓								
**Interaction Network Ontology (INO)**	1		✓														
**InterPro**	4	✓			✓	✓					✓						
**INTACT**	1	✓															
**iPTMnet**	1			✓													
**Jensen Lab Diseases**	1		✓														
**KEGG**	4		✓	✓	✓	✓											
**KFPT**	1									✓							

KG SOURCES

	Total # KG per source	Monarch KG	RTX-KG2	ROBOKOP	Bioteque	SPOKE	GenomicKB	HetioNet	clinical KG	Petagraph	DrugMechDB	PrimeKG	NCATS Gard	HRA-KG	EmBiology	PharmKG	GNBR
**KinAce**	1			✓													
**LabeledIn**	2					✓		✓									
**LIFEDB**	1	✓															
**LINCS 100**	4			✓		✓		✓		✓							
**LungMAP Human Anatomy (LMHA)**	1													✓			
**Logical Observation Identifiers Names and Codes (LOINC)**	1		✓														
**Mammalian Phenotype Ontology**	2	✓											✓				
**Mass Spectrometry Ontology**	1								✓								
**Mayo Clinic**	1											✓					
**MAXO**	1	✓															
**MEDI**	2					✓		✓									
**Medication reference terminology (MED-RT)**	1		✓														
**MedGen**	1												✓				
**MedLine**	2					✓		✓									
**MEDLINE Plus**	1		✓														
**MetabolomicsWorkbench**	1			✓													
**MetaCyc**	1					✓											
**Metathesaurus**	1		✓														
**MeSH**	5		✓			✓		✓			✓		✓				
**MGI**	3	✓		✓						✓							

KG SOURCES

	Total # KG per source	Monarch KG	RTX-KG2	ROBOKOP	Bioteque	SPOKE	GenomicKB	HetioNet	clinical KG	Petagraph	DrugMechDB	PrimeKG	NCATS Gard	HRA-KG	EmBiology	PharmKG	GNBR
miRBase	1		✓														
Molecular Interactions Controlled Vocabulary (MI)	1		✓														
Molecular Interactions Ontology	1								✓								
Molecular Signatures Database (MSigDB)	1									✓							
MonarchKG	1			✓													
MONDO	5	✓	✓	✓								✓	✓				
MotifMap	1						✓										
MPATH	1	✓															
Mouse gene-to-phenotype mappings (MPMGI)	1									✓							
MTBBASE	1	✓															
MutationDs	1								✓								
NCBI Gene	2	✓	✓														
NCBI Taxonomy	3		✓			✓					✓						
NCBO BioPortal	2									✓			✓				
National Cancer Institute (NCI)	1		✓														
National Drug data File	1		✓														
National drug data file-reference terminology	1		✓														
Neuro Behavior Ontology (NBO)	2	✓	✓														
NTNU-SB	1	✓															
OBA	1	✓															

KG SOURCES

	Total # KG per source	Monarch KG	RTX-KG2	ROBOKOP	Bioteque	SPOKE	GenomicKB	HetioNet	clinical KG	Petagraph	DrugMechDB	PrimeKG	NCATS Gard	HRA-KG	EmBiology	PharmKG	GNBR
**OBO Foundry**	2		☑							☑							
**Offsides**	1				☑												
**OMIM**	6	☑	☑	☑		☑							☑			☑	
**Omnipath**	1				☑												
**OncoKB**	1								☑								
**Ontology of Genes and Genomes**	1												☑				
**Open Targets**	1				☑												
**Orphanet**	5	☑	☑	☑								☑	☑				
**PANTHER**	2	☑		☑													
**Parkinsonsuk-UCL**	1	☑															
**PathBank**	1		☑														
**Pathway Interaction Database**	2					☑		☑									
**PATRIC**	1					☑											
**PathoPhenoDB**	1					☑											
**PATO**	1	☑															
**PDSP**	1			☑													
**PharmacoDB**	1				☑												
**PharmGKB**	1															☑	
**Pharos**	1			☑													
**Phenio**	1	☑															

KG SOURCES

	Total # KG per source	Monarch KG	RTX-KG2	ROBOKOP	Bioteque	SPOKE	GenomicKB	HetioNet	clinical KG	Petagraph	DrugMechDB	PrimeKG	NCATS Gard	HRA-KG	EmBiology	PharmKG	GNBR
**Phenotype and Trait Ontology (PATO)**	2		✓										✓				
**PHI-BASE**	0																
**PhosphoSitePlus**	2			✓					✓								
**Physician data query**	1		✓														
**PINC**	1	✓															
**Pfam**	3					✓			✓		✓						
**PomBase**	1	✓															
**PPI**	1											✓					
**PREDICT**	2					✓		✓									
**PRISM**	1				✓												
**PROGENy**	1															✓	
**ProtCID**	1					✓											
**Protien Modification Ontology**	2		✓						✓								
**Protein Ontology**	3	✓									✓		✓				
**Provisional Cell Ontology (PCL)**	1													✓			
**Psychological index terms**	1		✓														
**Reactome**	8	✓	✓	✓	✓	✓		✓			✓	✓					
**Reaxy**	1														✓		
**RefSeq**	2								✓	✓							
**RegNetwork**	1						✓										

KG SOURCES

	Total # KG per source	Monarch KG	RTX-KG2	ROBOKOP	Bioteque	SPOKE	GenomicKB	HetioNet	clinical KG	Petagraph	DrugMechDB	PrimeKG	NCATS Gard	HRA-KG	EmBiology	PharmKG	GNBR
**Relation Ontology (RO)**	3	✓	✓											✓			
**RepoDB**	1				✓												
**Repohub**	1				✓												
**RGD**	2	✓		✓													
**RHEA**	1	✓															
**RNAcentral**	2	✓					✓										
**ROSLIN-Institute**	1	✓															
**RxNorm**	1		✓														
**SCREEN**	1						✓										
**Sequence Ontology**	1	✓															
**SemMedDB**	1		✓														
**SGD**	2	✓		✓													
**SIDER**	7			✓	✓	✓		✓	✓			✓				✓	
**SIGNOR**	2			✓					✓								
**SMPDB**	2		✓						✓								
**SNOMED-CT**	1								✓								
**STARGEO**	2					✓		✓									
**STITCH**	1								✓								
**STRING**	6	✓		✓	✓	✓			✓	✓							
**SYNGO**	1	✓															

KG SOURCES

	Total # KG per source	Monarch KG	RTX-KG2	ROBOKOP	Bioteque	SPOKE	GenomicKB	HetioNet	clinical KG	Petagraph	DrugMechDB	PrimeKG	NCATS Gard	HRA-KG	EmBiology	PharmKG	GNBR
**SYNGO-UCL**	1	✓															
**SYSCILLIA-CCNET**	1	✓															
**Thesaurus**	1												✓				
**TISSUES**	5			✓	✓	✓		✓	✓								
**TTD**	1															✓	
**Uberon**	7	✓	✓			✓	✓	✓			✓			✓			
**UBKG**	1									✓							
**UniChem**	1		✓														
**Units Ontology**	1								✓								
**UniProt**	7	✓	✓	✓		✓			✓	✓	✓						
**UMLS**	3		✓							✓		✓					
**UPHENO**	1	✓															
**Vasculature Common Coordinate Framework (VCCF)**	1													✓			
**Veterans Affairs National Drug File (VANDF)**	2		✓										✓				
**Wbbt**	1	✓															
**WBLS**	1	✓															
**WBPhenotype**	1	✓															
**WikiPathways**	2					✓		✓									
**Wikipedia articles**	1										✓						
**WormBase**	1	✓															

KG SOURCES

	Total # KG per source	Monarch KG	RTX-KG2	ROBOKOP	Bioteque	SPOKE	GenomicKB	HetioNet	clinical KG	Petagraph	DrugMechDB	PrimeKG	NCATS Gard	HRA-KG	EmBiology	PharmKG	GNBR
**XAO**	1	☑															
**XenBase**	1	☑															
**XPO**	1	☑															
**Yubio Lab**	1	☑															
**Zfin**	2	☑		☑													
**Zebrafish Phenotype Ontology**	1	☑															
**Zebrafish Anatomy and development Ontologies**	1	☑															
**ZFS**	1	☑															
**4DNucleome**	2						☑			☑							
**Other - NLP/text mining**	4			☑							☑				☑		☑
**Other**	4				☑		☑				☑				☑		
